# Risk Factors for Postoperative Pulmonary Complications after Abdominal Surgery

**DOI:** 10.3889/oamjms.2016.059

**Published:** 2016-05-22

**Authors:** Nertila Kodra, Vjollca Shpata, Ilir Ohri

**Affiliations:** 1 *University Hospital Center “Mother Teresa”, Tirana, Albania*; 2 *Faculty of Medical Technical Sciences, University of Medicine, Tirana, Albania*; 3 *Department of Anesthesia and Intensive Care, Faculty of Medicine, University of Medicine, Tirana, Albania*

**Keywords:** postoperative, pulmonary, complications, risk factor, abdominal surgery

## Abstract

**BACKGROUND:**

Incidence of postoperative pulmonary complications (PPC) in patients undergoing non-cardiothoracic surgery remains high and the occurrence of these complications has enormous implications for the patient and the health care system.

**AIM:**

The aim of the study was to identify risk factors for PPC in patients undergoing abdominal surgical procedures.

**MATERIALS AND METHODS:**

A prospective cohort study in abdominal surgical patients, admitted to the emergency and surgical ward of the UHC of Tirana, Albania, was conducted during the period: March 2014-March 2015. We collected data on the occurrence of a symptomatic and clinically significant PPC using clinical, laboratory, and radiology data. We evaluated the relations between PPCs and various pre-operative or intra-operative factors to identify risk factors.

**RESULTS:**

A total of 450 postoperative patients admitted to the surgical emergency and surgical ward were studied. The mean age were 59.85 ±13.64 years with 59.3% being male. Incidence of PPC was 27.3% (123 patients) and hospital length of stay was 4.93 ± 4.65 days. Length of stay was substantially prolonged for those patients who developed PPC (7.48 ± 2.89 days versus 3.97± 4.83 days, p < 0.0001. PPC were identified as risk factors for mortality, OR: 21.84; 95% CI: 11.66-40.89; P < 0.0001. The multivariate regression analysis identified as being independently associated with an increased risk of PPC: age ≥ 65 years (OR 11.41; 95% CI: 4.84-26.91, p < 0.0001), duration of operation ≥ 2.5 hours (OR 8.38; 95% CI: 1.52-46.03, p = 0.01, history of previous pulmonary diseases (OR 11.12; 95% CI: 3.28-37.65, P = 0.0001) and ASA > 2 (OR 6.37; 95% CI: 1.54-26.36, P = 0.01).

**CONCLUSION:**

We must do some efforts in reducing postoperative pulmonary complications, firstly to identify which patients are at increased risk, and then following more closely high-risk patients because those patients are most likely to benefit.

## Introduction

Incidence of postoperative pulmonary complications in patients undergoing non-cardiothoracic surgery remains high [1] and the occurrence of these complications has enormous implications for the patient and the health care system [2].

Postoperative pulmonary complications (PPC) occur in 2% to 40% of patients and are associated with increased morbidity, mortality, and length of hospital stay [2-4].

In our country, there is no study about the incidence and risk factors for PPC. Previous international studies demonstrated that the majority of risk factors for PPC can be intervened and improved, [5, 6] so identifying which patients are at increased risk, we can reduce postoperative pulmonary complications. Studies have shown that pulmonary complications make the second most serious morbidity after cardiovascular event, one in four deaths occurring within a week of surgery is related to pulmonary complications [7]. Already has been explored that identifying perioperative risk factors of PPC is an important step toward improving quality of care in surgical patients [8-10]. The National Surgical Quality Improvement Program that compared hospitalization costs and length of stay among patients with various postoperative complications found that pulmonary complications were by far most costly [4]. So identifying patients at risk for pulmonary complications and developing a strategy to reduce the risk is clearly worthwhile [11].

The aim of the study was to identify risk factors for postoperative pulmonary complications in patients undergoing abdominal surgical procedures.

## Materials and Methods

### Study design and patient population

A prospective cohort study in abdominal surgical patients, admitted to the emergency and surgical ward of the University Hospital Centre “Mother Teresa” of Tirana, Albania, was conducted during the period: March 2014-March 2015. Throughout this period, consecutive patients aged > 18 years that underwent abdominal surgery and stayed in the hospital for more than 24 hours were enrolled in the study. The exclusion criteria: pregnancy, patients with preoperatively intubated trachea, and patients who underwent thoracoabdominal incision.

This study was approved by the Ethics Committee of the University of Medicine, Tirana, Albania. It has been performed in accordance with the ethical standards displayed in the 1964 Declaration of Helsinki and its later amendments. Data were made anonymous for analysis.

#### Demographic and medical information

Demographic and medical information including sex, age, date of hospital admission, diagnosis, and type of hospital admission (emergency or elective) ASA (American Society of Anesthesiologists) class were collected [12]. The ASA classification is a general index of overall morbidity that ranges from class 1 (normal healthy patient) to class 5 (moribund patient who is not expected to survive without the operation) and class 6 (declared brain-dead patient whose organs are being removed for donor purposes). We recorded history of chronic obstructive pulmonary disease (COPD) and history of asthma as preexistence of pulmonary diseases.

### Postoperative pulmonary complications

We collected data on the occurrence of a symptomatic and clinically significant postoperative pulmonary complication using clinical, laboratory, and radiology data. These included: respiratory failure requiring mechanical ventilation, pneumonia. [13], macroscopic atelectasis (by chest radiography), and pneumothorax, mass pleural effusion requiring percutaneous intervention.

Length of hospital stay was measured in days, from the day of hospital admission to the time of discharge or death.

Data were collected prospectively to determine ICU admission and stay, and mortality.

We evaluated the relations between PPCs and various pre-operative or intra-operative factors to identify risk factors.

### Statistical analysis

Continuous variables were presented as the mean ± SD (standard deviation). Categorical variables were expressed as actual numbers (n) and percentages (%). Linear and logistic regression was conducted to test the relation between the postoperative pulmonary complications (PPC) and other variables. Multivariate analysis was conducted using a logistic regression model in which “postoperative pulmonary complication” was used as the dependent variable. Only the variables associated with PPC that yielded *P*-values lower than 0.10 in the initial analysis were used in the stepwise logistic regression analysis. Odds ratios (OR) and 95% confidence intervals (95% CIs) were used to estimate the association between PPC and other variables.

Also, this model was used to analyze the effect of PPC on the length of hospital stay and mortality. Chi-square analysis was applied to compare frequencies between subgroups, and Student’s *t-*tests, or non-parametric tests when necessary, were employed for quantitative variable analysis.

Statistical significance was considered at the level of p ≤ 0.05. All tests were two tailed. SPSS 15.0 statistical package used to analyze the data.

## Results

A total of 450 postoperative patients admitted to the surgical emergency and surgical ward were studied. The mean age was 59.85 ± 13.64 years (range: 27-86) with 59.3% (n = 267) being male.

**Figure 1:**
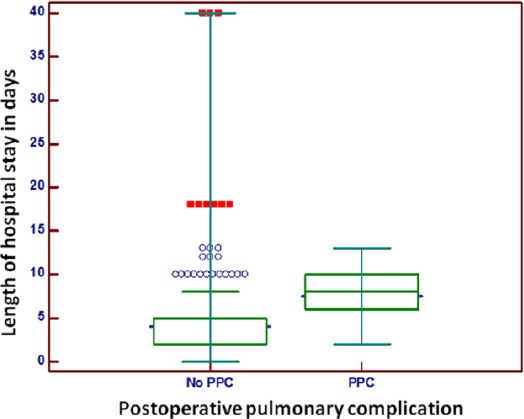
*Length of hospital stay in days according to the developing of postoperative pulmonary complications (PPC)*.

Incidence of PPC was 27.3% (123 patients) and hospital length of stay was 4.93 ± 4.65 days. Length of hospital stay was substantially prolonged for those patients who developed postoperative pulmonary complications (7.48 ± 2.89 days versus 3.97 ± 4.83 days, p < 0.0001, (Figure 1).

**Figure 2:**
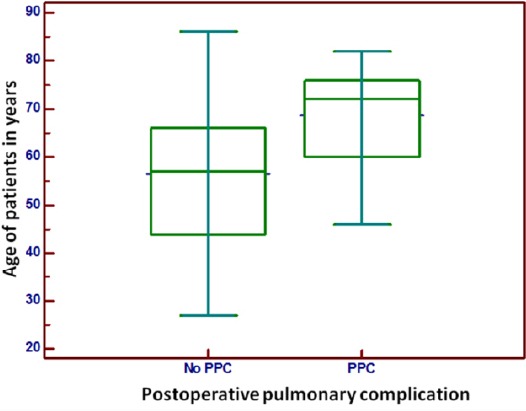
*Incidence of postoperative pulmonary complications (PPC) according to the age of patients in years*.

Average age was significantly higher in those with PPC compared to those without (64.93 ± 13.87 and 59.68 ± 15.37, respectively) (Figure 2).

**Figure 3:**
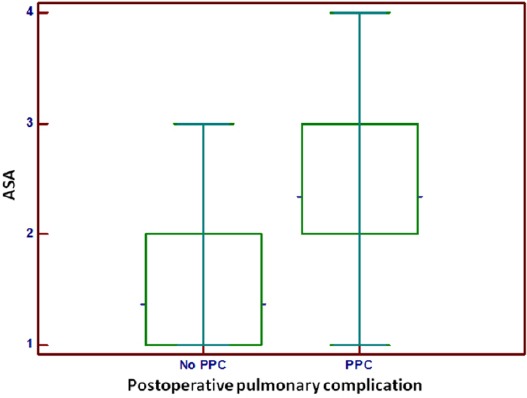
*Incidence of postoperative pulmonary complications (PPC) according to the ASA class of the patients preoperatively*.

Seventy five (75) patients (16.7%) were deemed by the anesthesiologist to be American Society of Anesthesiologists (ASA) class 3 or 4, with increasing of ASA class level was increased the incidence of PPC (Table 1 and Figure 3).

**Table 1:** Postoperative pulmonary complications according to the level ASA class of the patients

	ASA class 1 No of patients	ASA class 2 No of patients	ASA class 3 No of patients	ASA class 4 No of patients
Patients without PPC	219	96	12	0

Patients with PPC	24	36	60	3

The results of univariate analyses are outlined in Table 2. 90 patients (73.17% of the patients who developed postoperative pulmonary complications) were transferred to the Intensive Care Unit. Hospital mortality was 17.3% (n = 78) and 60 patients (48.78%) of the patients who developed postoperative pulmonary complications died.

**Table 2:** Results of univariate analysis for significant risk factors for PPC

Variable	No. of patients (%)	No. of patients with particular finding	OR (95% CI)	P Value
Male gender	267 (59.3%)	84	1.69 (1.09-2.62)	0.018

Age ≥ 65 yr	180 (40%)	87	6.08 (3.85-9.60)	< 0.0001

Emergency admission	63 (14%)	63	687.47 (41.96-11262.8)	< 0.0001

Current smoking	153 (34%)	60	2.39 (1.56-3.67)	0.0001

Presence of malignant disease	138 (30.7%)	66	4.10 (2.63-6.37)	< 0.0001

Preexistence of pulmonary diseases	60 (13.3%)	51	25.02 (11.78-53)	< 0.0001

ASA class > 2	75 (16.7%)	63	27.56 (14.01-54.2)	< 0.0001

Estimated bleeding during surgical procedure ≥ 500 mL	63 (14%)	39	5.86 (3.33-10.29)	< 0.0001

Pre-operative blood transfusion	33 (7.3%)	24	8.56 (3.85-19.03)	< 0.0001

Intra and post-operative blood transfusion	72 (16%)	45	6.41 (3.74-10.98)	< 0.0001

Duration of operation ≥ 2.5 hours	162 (36%)	90	9.65 (5.99-15.56)	< 0.0001

Perioperative nasogastric tube	358 (79.6%)	118	8.55 (3.38-21.63)	< 0.0001

PPC: post-operative pulmonary complication; OR: odds ratio; CI: confidence interval

PPC were identified as risk factors for mortality, OR: 21.84; 95%CI: 11.66-40.89; P < 0.0001. The multivariate regression analysis identified as being independently associated with an increased risk of postoperative pulmonary complications: age ≥ 65 years (OR 11.41; 95% CI: 4.84-26.91, p < 0.0001), duration of operation ≥ 2.5 hours (OR 8.38; 95%CI: 1.52-46.03, p = 0.01, history of previous pulmonary diseases (OR 11.12; 95%; CI: 3.28-37.65, P = 0.0001) and ASA class 3, or ASA class 4 (OR 6.37; 95% CI: 1.54-26.36, P = 0.01).

## Discussion

The present study found that there was high incidence of PPC. This incidence is comparable with the incidence of PPC in high risk patients, because we excluded patients that stayed < 24 hour in the hospital. However, respiratory complications occur within 48-72 h following surgery [14].

Perioperative variables associated with postoperative pulmonary complications were: male gender, age ≥ 65 years, emergency admission, current smoking, presence of malignant disease, preexistence of pulmonary diseases, estimated bleeding during surgical procedure ≥ 500 mL, perioperative blood transfusion, duration of operation ≥ 2.5 hours and perioperative placement of nasogastric tube. However, only four of these variables were independently associated with increased risk after multivariate analysis: age ≥ 65 years, duration of operation ≥ 2.5 hours, history of previous pulmonary diseases and ASA > 2.

As it has been reported, we found significant associations between age, history of previous pulmonary disease and postoperative pulmonary complications [9, 14]. Although those are unmodifiable risk factors, a post-operative meticulous management can prevent progression to severe complications in these patients.

Our findings showed that ASA > 2 is a risk factor [15]. Several studies have shown that duration of surgery is an independent risk predictor for PPC, and the present study confirmed this finding [2, 16-18].

In our study we found that a previous pulmonary disease is a risk factor for PPC. Our findings are consistent with other studies that have shown a correlation between COPD, asthma and PPC [17].

PPC occur more often in males than in females, a possible explanation being that men tend to breathe more with their diaphragm and women more with their thorax; thus when the movements of the diaphragm are restricted after upper abdominal operations the males suffer more from lack of expansion of the lungs [19].

In our clinical practice we use routine nasogastric decompression. Our study, like other studies, [2, 20, 21] showed that placement of a nasogastric tube perioperatively increases the risk of pulmonary complications. These findings supports the selective nasogastric decompression only when it is judged to be necessary on clinical grounds [2], leading to the reduction of PPC [6].

The present study showed that smoking history correlates with pulmonary complications. [9, 14, 16, 22]. The presence of PPC lead to prolonged hospital stay and increased mortality rate, as in other studies [2, 23].

One limitation of the study was that we didn’t evaluate the risk factors for PPC independently for each type of surgery.

In conclusion, the present study found that there was high incidence of PPC which increased hospital length of stay and hospital mortality in the abdominal surgery. We identified twelve factors associated with the occurrence of PPC. We must do some efforts in reducing postoperative pulmonary complications, firstly to identify which patients are at increased risk, and then following more closely high-risk patients because those patients are most likely to benefit. We have demonstrated some perioperative factors that can be used to identify patients at increased risk for postoperative pulmonary complications after abdominal surgery.
